# The BPD trio? Interaction of dysregulated PDGF, VEGF, and TGF signaling in neonatal chronic lung disease

**DOI:** 10.1186/s40348-017-0076-8

**Published:** 2017-11-07

**Authors:** Prajakta Oak, Anne Hilgendorff

**Affiliations:** 1 Comprehensive Pneumology Center, University Hospital of the University of Munich and Helmholtz Zentrum Muenchen, Munich, Germany; 2 Department of Neonatology, Perinatal Center Grosshadern, Ludwig‐Maximilians University, Munich, Germany; 3 Center for Comprehensive Developmental Care, Dr. von Haunersches Children’s Hospital University, Hospital Ludwig‐Maximilians University, Munich, Germany

## Abstract

The development of neonatal chronic lung disease (nCLD), i.e., bronchopulmonary dysplasia (BPD) in preterm infants, significantly determines long-term outcome in this patient population. Risk factors include mechanical ventilation and oxygen toxicity impacting on the immature lung resulting in impaired alveolarization and vascularization. Disease development is characterized by inflammation, extracellular matrix remodeling, and apoptosis, closely intertwined with the dysregulation of growth factor signaling. This review focuses on the causes and consequences of altered signaling in central pathways like transforming growth factor (TGF), platelet-derived growth factor (PDGF), and vascular endothelial growth factor (VEGF) driving these above indicated processes, i.e., inflammation, matrix remodeling, and vascular development. We emphasize the shared and distinct role of these pathways as well as their interconnection in disease initiation and progression, generating important knowledge for the development of future treatment strategies.

## Background

Neonatal chronic lung disease (nCLD), commonly known as bronchopulmonary dysplasia (BPD), is a frequent complication in very low birth weight preterm infants receiving mechanical ventilation with oxygen-rich gas (MV-O_2_) [1]. In this high-risk patient population, up to 30% of the infants born at less than 30 weeks of gestation (gestational age, GA) develop BPD [2]. It is associated with significant long-term pulmonary and neurologic sequelae persisting into adulthood [3, 4]. Clinically, BPD is defined by the need for supplemental oxygen or ventilator support at day 28 of life (mild BPD) or at 36 weeks gestational age (moderate and severe BPD) [5]. Both, shear stress as well as oxygen toxicity, are known to negatively impact development of the gas exchange area of lungs resulting in impaired structure and function of both vessels and alveoli, critically driven by an inflammatory response and significant extracellular matrix (ECM) remodeling [5]. Cause and consequence of these pathophysiologic processes is the dysregulation of central signaling pathways that play a critical role in both normal lung development as well as disease initiation and progression. In comparison to the understanding of a single pathway, knowledge about the interplay of these important regulators of disease pathophysiology is of significant importance for the development of future preventive and causative treatment strategies.

The review addresses important aspects of the crosstalk between key signaling pathways linking alveolar and vascular development with lung apoptosis and matrix remodeling [6]: part one of the review will focus on the platelet-derived growth factor (PDGF) as a critical driver of alveolar septation, whereas part two will address the role of the vascular endothelial growth factor (VEGF) as a key regulator in vascular development. Finally, a chapter reviewing the role of the transforming growth factor (TGF) β, known for its impact on apoptosis, matrix remodeling, and inflammatory processes in the developing lung, will connect important aspects discussed on behalf of the abovementioned signaling pathways in a disease-relevant context [7].

### The role of PDGF signaling in (patho)-physiologic lung development

The saccular and alveolar phase of lung development is characterized by the process of secondary septation, critically driven by the migration of the PDGF receptor (PDGF-R) α expressing myofibroblast (MFB) towards the tip of the septal crest, and the deposition of elastin at this position providing the scaffold for the developing alveoli [8–10].

The important role of PDGF-Rα signaling in lung development is shown by studies performed on homozygous knockout mice for PDGF-A as well as PDGF-Rα. Very few mice pups from homozygous knockout of PDGF-A escape in utero death caused by distorted lung development, leading to an emphysematous-like phenotype in newborn mice [11]. Simultaneously, mice lacking the respective receptor only survive until early embryonic stages as a result of respiratory failure due to a simplified pulmonary structure with a decrease in secondary crests and a subsequent increase in alveolar size, thereby resembling the phenotype observed in neonatal rats treated with a PDGF-Rα antagonist [12, 13].

The failure in alveologenesis in PDGF-Rα knockout mice is assigned to the reduced migration of PDGF-Rα **+** alveolar smooth muscle progenitor cells, i.e., MFBs towards the secondary crest, confirming the important role of PDGF-Rα signaling in MFBs migration [14, 15]. Along these lines, Popova et al., demonstrate a reduced chemotaxis of pulmonary mesenchymal cells isolated from BPD patients with reduced mRNA levels for PDGF-Rα [16], that is in concert with studies performed on ventilated newborn mice and lamb connecting dysregulated PDGF-A and PDGF-Rα signaling to disease development [17–20]. In a three-dimensional study performed in lungs of hyperoxia-exposed mice, the dislocation of α-SMA positive MFBs corresponds with a disruption of the pulmonary elastin network [21]. As shown in patients with BPD and further evaluated by studies in newborn transgenic mice, the breakdown and pathologic remodeling of the elastic network characterizes alveolar branching arrest, increased airspace size, and simplification of the gas exchange area, all contributing to significant in long-term complications [10, 22–26].

In order to generate a deeper understanding of the interplay between early more generalized disease processes with cell-specific pathways and identify potential treatment targets, we will address the specific interaction of the pro-inflammatory and pro-apoptotic cytokine TGF-β and its interplay with the PDGF and VEGF pathway, linking the characteristic inflammatory response in BPD development to critical drivers of alveolarization and vascular development (see Chapter III TGF-β signalling enhancing pulmonary injury in neonates of this review).

### Disturbed vascular growth factor signaling in the injured neonatal lung

Studies in premature rats undergoing postnatal hyperoxia exposure demonstrate a reduction in pulmonary VEGF-R2 expression, a receptor on endothelial cells mediating their survival and function [27]. Animal models of BPD using MV-O_2_ to trigger disease development lead to defective vasculature and alveolar structure accompanied by decreased VEGFA/VEGF-R2 signaling [28, 29]. According to the “vascular hypothesis of lung development,” endothelial cell signaling is a crucial pre-requisite for the formation of the alveolar structure [30]. In line with this, the use of a VEGF receptor inhibitor in animal models of BPD hinders both pulmonary angiogenesis and alveolar development, resulting in reduced lung weight [31–33]. In this process, VEGF holds an important role impacting on endothelial and epithelial cell signaling [34, 35]. Indicating the severely disturbed signaling process in BPD patients, studies in broncho-alveolar lavage fluid of BPD patients show significantly reduced VEGF levels [36], in line with genetic polymorphism in the VEGF gene associated with disease development [37]. Disturbed VEGF signaling is paralleled by increased levels of the anti-angiogenic proteins thrombospondin-1 and endoglin together with decreased expression levels of the pro-angiogenic factor Tie-2 [38, 39].

Retinopathy of prematurity (ROP), a condition in preterm infants characterized by comparable complications in vascular development, is associated with reduced levels of insulin-like growth factor (IGF)-1 that in turn abrogates VEGF-A expression through the MAPK pathway leading to endothelial cell apoptosis [40]. Coincidentally, low pulmonary IGF-1 expression can be considered as a driver of disrupted formation of the capillary bed in the developing lung undergoing postnatal injury in the same cohort of high-risk preterm infants [41].

Seemingly in contrast, pulmonary overexpression of VEGF-A in newborn mice leads to the development of a simplified lung structure with fewer and larger alveoli when the respective mice are exposed to hyperoxia [42]. These findings are in line with treatment studies using VEGF-A that failed to rescue the lung from postnatal injury but resulted in severe capillary leakage [43]. These findings further indicate the critical regulation of VEGF expression throughout lung development in concert with the complex crosstalk of other growth factors. The elevation of VEGF levels upon initiation of postnatal injury—as observed in some studies in BPD patients—may indicate a rescue mechanism underlining the essential role of VEGF-A in endothelial cell survival including mobilization of endothelial progenitor cells [44–46]. Another source for the (transitory) increase in pulmonary VEGF expression upon injury is monocytes/macrophages recruited to the injured lung [47].

With respect to its upstream regulation, hypoxia-inducible factor [9] plays an important role as it induces pulmonary VEGF expression [48]. Accordingly, the expression of a mutant HIF gene leads to the development of postnatal respiratory distress together with a simplified alveolar structure in the lungs of fetal mice. The outlined results point towards a role for the co-regulation of HIF and VEGF in BPD pathophysiology side by side with other potential regulators of VEGF signaling such as nuclear factor-ƙB, where abrogation of its expression induces apoptosis, simplifies alveolar structure, and reduces pulmonary capillary density in neonatal mice in concert with a significant reduction of VEGF-R2 expression [49].

Knowledge about reduced expression of VEGF-A and VEGF-R2 in ventilated neonatal mice in association with a decrease in PDGF-A expression first indicated a potential co-regulation of these pathways during disease development [19]. In our own studies, we therefore comprehensively investigate the specific interaction of these two signaling pathways and show that abrogation of PDGF-Rα signaling leads to reduced VEGF-A production by the MFB, thereby leading to endothelial cell apoptosis in ventilated newborn mice. Using transgenic mice in a pre-clinical model and detailed in vitro studies, we not only establish a causal relationship between the two pathways through the lung MFB but, at the same time, show that PDGF treatment is able to successfully restore VEGF signaling in the newborn mouse lung ([50] (in press). This first step towards the development of new treatment concepts has to be pursued further in order to address other co-regulated pathways in this context.

### TGF-β signaling enhancing pulmonary injury in neonates

The TGF-β protein is secreted into the extracellular matrix (ECM) in its inactive form bound to latent TGF-β binding proteins (LTBPs) [51]. In turn, binding of LTPBs to other ECM proteins like fibrillin 1 and 5 facilitates elastogenesis in the developing lung [51]. Next to its secretion by inflammatory cells, the release of TGF-β is observed as a consequence of ECM remodeling [52, 53]. As a consequence, both, the phosphorylation of SMAD proteins as well as the upregulation of connective tissue growth factor (CTGF), are associated with the activation of the TGF-β pathway [54]. In addition to the important role of TGF-β for normal lung development, studies in different animal models of BPD confirm elevation of TGF-β expression levels and activation of its associated pathway as an important part of disease pathophysiology [24, 28, 55, 56]. Supporting the relevance of these findings from experimental studies, clinical studies show increased expression as well as activation of TGF-β, an inflammatory cytokine and growth factor in BALF obtained from ventilated preterm infants [57].

Increase in pulmonary TGFβ expression stems from the characteristic inflammatory response preceding most of the BPD-relevant pathopysiology [58]. The influx of inflammatory cells, i.e., neutrophils and monocytes, into the injured lung during disease development is well described in infants with BPD [59, 60]. Consequently, the recruited monocytes and macrophages are the most important source of TGF-β [61] together with a simultaneous increase in secreted cytokines such as interleukin (IL-)1β, IL-6, IL-8, tumor necrosis factor (TNF)-α, monocyte chemo-attractant proteins (MCP) 1 to 3, and the macrophage inflammatory proteins (MIP) 1a and 1b in preterm infants with evolving BPD [57, 62, 63]. Pro-inflammatory factors, in turn, provoke the development of pulmonary edema in ventilated preterm infants and lambs [64–66]. The pro-inflammatory response is typically associated with decreased levels of their anti-inflammatory counterparts, i.e., IL-10, IL-4, IL-12, and IL-13 or the IL-1 receptor antagonist [67–70].

Remodeling of the ECM not only leads to the release of TGF-β [36, 71, 72] but, at the same time, is a consequence of its increased expression and signaling. Both, BPD patients as well as animals with a BPD phenotype induced by postnatal hyperoxia, exhibit a TGF-β dependant upregulation of lysyl oxidases and transglutaminases—enzyme that aids in crosslinking of elastin, collagen, and fibronectin [73, 74]. The overexpression of TGF-β furthermore results in impaired branching morphogenesis, altered cellular composition, and ECM remodeling in neonatal mice [72]. In line with these findings, the use of a TGF-β neutralizing antibody rescues the alveolar and microvascular phenotype occurring in newborn mice exposed to hyperoxia [75].

A possible mechanism underlying the TGF-β-driven alveolar pathology is indicated by in vitro findings that show a reduction of PDGF expression by TGF β in adult pulmonary mesenchymal cells [76]. Heinzelmann et al. furthermore demonstrate a negative correlation between TGF-β stimulation and PDGF-Rα expression in adult human lung fibroblasts [77]. Translating these findings into the developing human lung, recent studies performed by Popova et al. display a downregulation in PDGF-Rα expression upon TGF-β treatment in lung mesenchymal-like cells (MSCs) obtained from BPD patients [16]. In support with the help of tailored in vitro assays employing primary mouse and human fibroblasts together with a unique mouse model of the disease, we confirm a causal relationship between reduced PDGF-Rα expression and increased TGF-β expression ([50] (in press). Not only does the increased TGF-β activation coincides with reduction in PDGF expression but also with a decrease in VEGF signaling in animal models employing MV-O_2_ to induce a BPD phenotype [22]. This finding is further supported by antibody blocking of TGF signaling leading to a VEGF-dependant improvement in microvascular structure and function [75]. Different effects attributed to TGF-β can play a causal role in this context: one, the induction of the CTGF by TGF-β as shown in airway smooth muscle cells facilitates the binding of VEGF to the ECM [78]; second, the bone morphogenic protein (BMP), part of the TGF-β superfamily, enhances secretion of the VEGF protein in murine proteoblastic KS483 cells [79]. This is in line with our data, showing a TGF-β-induced and PDGF-Rα-dependant regulation of VEGF expression in the injured neonatal lung ([50] (in press). TGF-α, a growth factor from the same cytokine family, is known for its role in cell proliferation, branching morphogenesis, and epithelial cell differentiation [80]. Accordingly, its overexpression in transgenic mice leads to pulmonary hypertension along with pathologic muscularization of small pulmonary arteries [81]. Likewise, FGF (fibroblast growth factor)-10 is expressed on mesenchymal cells and crucial for branching morphogenesis. The interaction of FGF-10 with PDGF and VEGF signaling and their role in BPD pathophysiology needs further investigation but holds promising value for the development of future treatment strategies [82, 83].

## Summary and conclusion

**Fig. 1:**
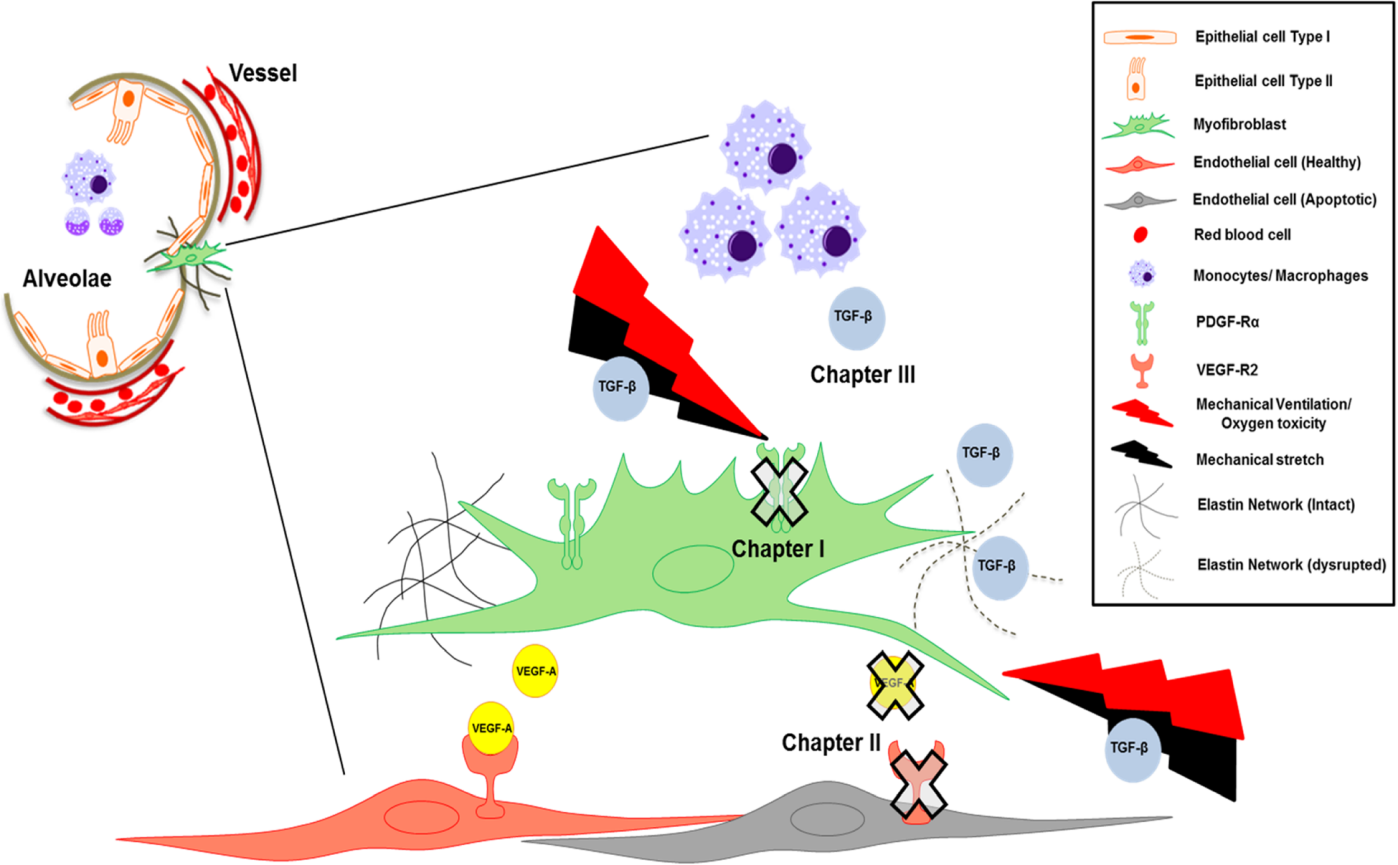
A schematic model depicting important aspects of our current understanding with respect to the crosstalk between the PDGF, VEGF and TGF pathway in the developing lung. Chapter I (The role of PDGF signalling in (patho)- physiologic lung development) emphasizes the importance of PDGF-Rα expressing myofibroblasts (MFBs) in alveolarization that is hindered by mechanical ventilation and stretch causing abrogation in the receptor leading to reduced migration and disrupted elastin deposition. As a consequence of its reduced downstream signaling, a reduction in VEGF-A is observed accelerating endothelial cell (EC) apoptosis. This is the link to chapter II (Disturbed vascular growth factor signalling in the injured neonatal lung) of the review showing interaction of PDGF to disrupted VEGF, a pro-survival growth factor for ECs. Chapter III (TGF-β signalling enhancing pulmonary injury in neonates) of the review focuses on the aspect of upregulated TGF-β in injured lung that can be attributed to increased influx of monocytes and macrophages. The increased TGF-β expression and signaling interferes with PDGF-Rα and VEGF-A signaling hence affecting survival and function of MFBs and ECs.

The multifactorial pattern of BPD requires a detailed understanding of different underlying signaling pathways, their critical crosstalk and the impact on disease pathophysiology. Figure 1 gives a schematic overview with respect to the interconnection of TGF activation, myofibroblast (PDGF) and endothelial cell (VEGF) signaling, and its impact on ECM remodeling eventually leading to defective alveolar and microvascular development. Deeper knowledge may reveal treatment options targeting different disease-relevant processes through one or two key processes.
